# Deletion of *Sod1* in Motor Neurons Exacerbates Age-Related Changes in Axons and Neuromuscular Junctions in Mice

**DOI:** 10.1523/ENEURO.0086-22.2023

**Published:** 2023-03-10

**Authors:** N. Pollock, P. C. Macpherson, C. A. Staunton, K. Hemmings, C. S. Davis, E. D. Owen, A. Vasilaki, H. Van Remmen, A. Richardson, A. McArdle, S. V. Brooks, M. J. Jackson

**Affiliations:** 1 Department of Musculoskeletal and Ageing Science, Institute of Life Course and Medical Sciences, University of Liverpool, and MRC-Versus Arthritis Centre for Integrated Research into Musculoskeletal Ageing (CIMA), Liverpool, L7 8TX, UK; 2 Molecular and Integrative Physiology, University of Michigan, Ann Arbor, 48109 MI; 3 Oklahoma Medical Research Foundation (OMRF), Oklahoma City, 73104, OK; 4 University of Oklahoma Health Science Center (OUHSC), Oklahoma City, 73104, OK

**Keywords:** axon, motor unit, oxidative stress, skeletal muscle

## Abstract

Whole-body knock-out of Cu,Zn superoxide dismutase (Sod1KO) results in accelerated, age-related loss of muscle mass and function associated with neuromuscular junction (NMJ) breakdown similar to sarcopenia. In order to determine whether altered redox in motor neurons underlies this phenotype, an inducible neuron-specific deletion of Sod1 (i-mnSod1KO) was compared with wild-type (WT) mice of different ages (adult, mid-age, and old) and whole-body Sod1KO mice. Nerve oxidative damage, motor neuron numbers and structural changes to neurons and NMJ were examined. Tamoxifen-induced deletion of neuronal Sod1 from two months of age. No specific effect of a lack of neuronal Sod1 was seen on markers of nerve oxidation (electron paramagnetic resonance of an *in vivo* spin probe, protein carbonyl, or protein 3-nitrotyrosine contents). i-mnSod1KO mice showed increased denervated NMJ, reduced numbers of large axons and increased number of small axons compared with old WT mice. A large proportion of the innervated NMJs in old i-mnSod1KO mice displayed a simpler structure than that seen in adult or old WT mice. Thus, previous work showed that neuronal deletion of Sod1 induced exaggerated loss of muscle in old mice, and we report that this deletion leads to a specific nerve phenotype including reduced axonal area, increased proportion of denervated NMJ, and reduced acetyl choline receptor complexity. Other changes in nerve and NMJ structure seen in the old i-mnSod1KO mice reflect aging of the mice.

## Significance Statement

Sarcopenia is the age-related loss of muscle mass and function. It is a significant contributor to frailty and to increased falls in the elderly. While multifactorial, changes in redox status have been shown to have significant influence over neuromuscular aging, recent work suggests that changes in motor neurons may be the driving factor in muscle atrophy. The current study confirmed that a specific lack of Sod1 in the motor neuron causes significant alteration in axonal architecture and the neuromuscular junctions (NMJs), which can drive reduced muscle mass and function. Pinpointing early changes in motor neurons may provide therapeutic targets critical for maintaining muscle in the elderly.

## Introduction

The cross-sectional area (CSA) of skeletal muscle in humans is reduced by 25–30% and muscle strength by 30–40% by age 70 (Porter et al., 1995). The reduction in muscle mass and function with age is because of a decrease in the number of muscle fibers and atrophy and weakening of those remaining (Lexell et al., 1986, 1988; Brooks and Faulkner, 1988). The loss of muscle that occurs with aging occurs in parallel with loss of motor units in both humans and rodents (Campbell et al., 1973; Sheth et al., 2018) and is associated with a 25–50% reduction in the number of motor neurons (Tomlinson and Irving, 1977; Rowan et al., 2012; Piekarz et al., 2020). This appears to be because of selective loss of large fast α-motor neurons leading to an apparent increase in the proportion of Type I (slow twitch) or Type IIa muscle fibers that is particularly apparent in humans (Narici and Maffulli, 2010; Sonjak et al., 2019). Loss of innervation of individual fibers occurs in muscles with aging and we previously reported that ∼15% of individual muscle fibers in old mice are completely denervated and ∼80% of neuromuscular junctions (NMJs) showed some disruption (Vasilaki et al., 2016).

In order to examine the potential role of oxidative stress and redox changes in aging, we previously examined the effect of a lack of superoxide dismutase1 (Sod1) in whole-body knock-out mice (Sod1KO) and demonstrated an accelerated loss of muscle mass and function that is associated with a breakdown of NMJs (Jang et al., 2010; Larkin et al., 2011). Adult mice lacking Sod1 exhibit many features observed in old wild-type (WT) mice (>24 months) including loss of muscle force, altered mitochondrial function and an accumulation of structural alterations at NMJs. Subsequent studies examined the effects of specific deletion of Sod1 in muscle (mSod1KO; Sakellariou et al., 2018) or motor neurons (nSod1KO; Sataranatarajan et al., 2015), but both models exhibited only a mild muscle phenotype, while expression of human SOD1 specifically in neurons of Sod1KO model was found to “rescue” the phenotype such that no premature loss of muscle mass nor alterations in NMJ structure were observed (Sakellariou et al., 2014).

To address the possibility that embryonic neuronal Sod1 knock-down in the nSod1KO mice induced a compensatory effect, we have recently created an inducible motor neuron Sod1 KO (i-mnSod1KO) mouse model (Bhaskaran et al., 2020) to examine whether loss of Sod1 in motor neurons in adult-life affected age associated muscle wasting and weakness. These mice were found to have an age-related, accelerated loss of muscle mass which preceded that seen in old WT mice by six to eight months (Bhaskaran et al., 2020).

In the current study, we examine the effects of age and lack of Sod1 specifically in neurons on motor neuron number and explore the changes in nerve oxidation status as well as structural changes in axons and NMJs. Our aim was to define whether a neuron-specific lack of CuZnSOD would be sufficient to compromise neuronal oxidation, induce disruptions of neuronal and NMJ structure, and increase denervation.

## Materials and Methods

### Mice

The neuron specific inducible Sod1 knock-out mice (i-mnSod1KO) used in this study were tamoxifen-induced KOs (10 mg/ml tamoxifen administered at two and four months) generated as previous described at the Oklahoma Medical Research Foundation (OMRF; Bhaskaran et al., 2020). In order to understand the specific role of neuronal Sod1 in the premature loss of neuronal and muscle structure and mass, the i-mnSod1KO mice were compared with age-matched WT mice in three distinct groups: six to nine months (adult), 16–18 months (mid-age), and 24–29 months (old). The i-mnSod1KO mice were also compared with whole-body knock-out (Sod1KO) mice. Whole-body Sod1KO mice were bred at OMRF as previously described (Sakellariou et al., 2014). Mice were maintained under specific pathogen-free (SPF) conditions and shipped to the University of Liverpool or the University of Michigan, where they were maintained until required. Whole-body Sod1KO mice were also crossed with Thy1-CFP mice (The Jackson Laboratory) at the University of Liverpool to produce mice (Sod1KO-Thy1-CFP) expressing CFP in neurons to allow ready visualization of the nerve without the need for antibody staining. These mice had the phenotypic changes observed in founder Sod1KO mice. The SlickH Cre mouse used to generate i-mnSod1KO mice were originally developed as a model to delete genes in motor neurons, while also simultaneously labeling the neurons with YFP and hence all expressed YFP in motor neurons (Bhaskaran et al., 2020). Male and female mice were used throughout this study.

All mice were fed *ad libitum* on a standard laboratory diet, subjected to a 12/12 h light/dark cycle and maintained under SPF conditions. All experiments were performed in accordance with UK Home Office guidelines under the UK Animals (Scientific Procedures) Act 1986 and received ethical approval from the University of Liverpool Animal Welfare Ethical Review Body (AWERB).

### Functional data

Extensor digitorum longus (EDL) force data were gathered *in situ* as previously described (Brooks and Faulkner, 1988). Briefly, with the mouse under terminal anesthesia the whole EDL muscle was isolated, the distal tendon was severed and secured to the lever arm of a servomotor and the muscle was activated by stimulation of the peroneal nerve. Maximum isometric tetanic force (Po) was recorded and specific Po was calculated from cross-sectional area (CSA) of the EDL. Mice were subsequently killed by a schedule 1 procedure and sciatic nerves rapidly excised and used for biochemical analyses.

### Oxidation markers

#### Electron paramagnetic resonance

In order to define the activity of specific reactive oxygen species (ROS) in muscle and nerve an *in vivo* electron paramagnetic resonance spin probe protocol using 1-hydroxy-3-carboxy-2,2,5,5-tetramethylpyrrolidine (CPH; Noxygen) was used as previously described (McDonagh et al., 2016). The CPH probe was made up in PBS containing sodium diethyldithio-carbamate trihydrate (DETC; 5 μm) and desferoxamine methane sulfonate salt (DF; 25 μm) fresh before each procedure. Groups of mice were anaesthetized via inhalation of isoflurane (2%) and maintained at an appropriate level in this manner via nose cone throughout this procedure. Mice received a tail vein bolus injection (80 μl, 9 mg/kg body weight) followed by tail vein infusion (0.225 μg/kg body weight/min) of the probe for 2 h. The mice were killed by cervical dislocation and the sciatic nerve and gastrocnemius muscle were quickly excised, stored in Krebs buffer and frozen in liquid nitrogen. Samples were stored in liquid nitrogen in a vapor phase Dewar. In order to detect levels of the oxidized probe 3-carboxy-proxyl radical (CP), samples were placed into the finger Dewar of the Bruker e-scan benchtop EPR and scanned using the settings: microwave frequency 9.78 GHz, modulation frequency 86 kHz, modulation amplitude 6.15 G, gain 10^3^.

##### Western blot analysis

Following killing, sciatic nerve was quickly excised and snap frozen in liquid nitrogen. The frozen nerves were cryo-pulverized under liquid nitrogen and the resulting powder added to 40 μl RIPA buffer (Merck Millipore) 10× diluted with distilled water with protease inhibitors (Roche, complete, mini, EDTA-free protease inhibitor cocktail). Samples were sonicated on ice, centrifuged at 12,000 × *g* for 10 min at 4°C and the supernatant retained. Protein content was quantified by BCA assay. 20 μg of total protein per sample was separated by SDS-PAGE with 4% stacking and 12% resolving gels. Electrophoresis was conducted at 120 V for protein separation, followed by semi-dry transfer onto nitrocellulose membrane for 1.5 h at 300 mA (Bio-Rad Trans-Blot SD).

A Ponceau S (Sigma) stain was used to verify the effectiveness of protein transfer. The membrane was blocked using 5% fish skin gelatin (FSG) made up in Tris buffered saline with Tween blocking buffer (5% FSG TBS) for 1.5 h. The membranes were incubated with primary antibody against 3-nitrotyrosines (3-NTs; Abcam; 1:1000 dilution in 5% FSG TBS-T) overnight at 4°C. Duplicate gels were run to assess protein carbonyls using a kit which derivatizes proteins directly on the membrane following transblotting (Cell Biolabs). For this, proteins were transferred onto PVDF membrane, and derivatized as per manufacturer’s instructions, and subsequently incubated with primary antibody against DNP (Cell Biolab; 1:1000 dilution 5% FSG TBS-T) overnight at 4°C. All membranes were then washed with TBST, and incubated with appropriate secondary antibodies for 1 h (1:20,000 dilution in 5% FSG TBST with 0.01% SDS). Protein bands were visualized on a Licor Odyssey CLx imaging system.

### Sciatic nerve cross-sections

Sciatic nerves were dissected (from point of bifurcation at the knee up to the spinal column), placed onto a needle (to prevent curling and maintain orientation) and submerged in 10% neutral buffered formalin (NBF) overnight. Nerves were transferred into a 30% sucrose solution for cryo-protection for 5 d before embedding in colored OCT (Shandon Cryochrome) using cryomolds and freezing in liquid nitrogen cooled isopentane. Transverse sections (7 μm) of the nerve were cut on a cryostat (Leica CM1850). Sections were thawed, blocked in 5% goat serum for 1 h and stained using myelin protein zero (MPZ; 1 in 50 in 5% goat serum in PBS containing 1% Triton X-100; EPR20383, Abcam) overnight. Following incubation with Alexa Fluor-594 nm secondary slides were mounted in hard-set mountant with Dapi (Vectorlabs) and visualized on Zeiss LSM800 confocal microscope using 40× objective.

Image analysis was conducted using the free hand tool in ImageJ software (NIH). The axon and the outer edge of the stained myelin sheath of 100 axons were drawn around to provide area of the axon, area of the sheath and the distribution of axon sizes. G-ratios were calculated for each axon as the ratio of the inner axonal diameter to the total outer diameter including myelin sheath.

### Retrograde motor neuron labeling

In separate experiments, mice were anesthetized with 3% isoflurane/oxygen and the nerve branches to medial and lateral heads of the left gastrocnemius were exposed and transected. On the contralateral side the sciatic nerve was transected at the level of the mid-thigh. Motor neurons were retrograde labeled by placing sterile Gelfoam saturated with 10% FluoroRuby (Invitrogen) over the respective nerve stumps, suturing the Gelfoam in place and closing the incision sites for recovery. Postoperative analgesia was achieved by treating mice with Carprofen (5 mg/kg) daily for 3 d. Following schedule 1 cull, the lumbar regions of spinal cords were obtained by extrusion with 5 ml of PBS 7 d after the onset of labeling. Spinal cords were fixed overnight in 10% formalin at 4°C, rinsed in PBS and processed for optical clearing according to previously published work (Ertürk et al., 2012; Žygelytė et al., 2016). Labeled motor neurons were imaged using an Olympus FV1000 confocal microscope with the z-step size set at 4.5 μm. Z-stacks were visualized using ImageJ and the total number of labeled motor neurons were quantified with the use of the cell counter plug-in function while scrolling through z-stacks.

### NMJ analysis

NMJ analysis was conducted on EDL muscles with 20–60 NMJs per muscle analyzed. All mice utilized for these studies expressed a fluorescent protein in the nerves to allow ready visualization.

The EDL muscles were quickly excised following schedule 1 cull, pinned at roughly resting length onto sylgard plates (Fernell) and fixed in 10% NBF. Whole EDL muscles were incubated with α-bungarotoxin conjugated Alexa Fluor-647 nm (1:1000; Invitrogen) in PBS (1% Triton X-100) for 30 min. Muscles were imaged via confocal microscopy (Nikon A1). The components of the NMJs were visualized using 488 nm laser to excite the YFP in the i-mnSod1KO, 405 nm to excite CFP in Sod1KO-CFP and the WT litter mates. Images were collected via 10× or 60× water immersion lens (Nikon). Z-stacking (1- to 2-μm step) allowed for 3D evaluation of the NMJs.

Using NIS-elements software (Nikon) individual NMJs were identified and the ROI tool used to obtain the area of the muscle fiber that is occupied by individual NMJs. All NMJs were assessed and graded according to five separate measures of integrity and structure: (1) overlap of the presynaptic and postsynaptic regions (innervated, partial or denervated) and the (2) number of postsynaptic fragments (none, no fragmentation, <5, or >5 fragments), (3) the number of axonal sprouts and blebs, (4) the incidence of multi-innervated NMJs, and (5) the complexity of the NMJ. This classification ranged from “complex,” showing the typical structure seen in adult WT NMJ, to “basic,” in which the NMJ appeared as simple discs with little internal structure and resembled embryonic NMJs. One intermediate grade between complex and basic (“good”) was also used.

### Experimental design and statistical analysis

For all experiments the number of mice used are indicated in the associated figure legend. All statistics were conducted using GraphPad Prism 8 software, unless stated otherwise results are presented as mean ± SD.

For NMJ analysis, all NMJs present within a field of view were assigned an ID and counted/graded. Multiple 3D images per muscle were assessed and all measures were expressed as a percentage of the total number of NMJs in an image to correct for variation in the total number of NMJs seen in each image. For fragmentation, overlap and complexity two-way ANOVA with Tukey’s multiple comparisons was used, while for other measures one-way ANOVA with either Dunnett’s or Tukey’s multiple comparisons was used.

For Western blot analyses, it was only possible to load *n* = 1 onto each SDS gel for each group. In order to compare across gels the same muscle lysate was added to each and all results were normalized to this. The data were analyzed via one-way ANOVA. EPR data and force were also analyzed by one-way ANOVA. Significance values *p < 0.05, **p < 0.01, ***p < 0.001, ****p < 0.0001.

Wherever possible multiple analyses were undertaken on the nerve and muscle from each mouse. This was not possible for mice that underwent perfusion of spin probe for EPR analysis, or used for retrograde labeling, where separate mice were examined.

## Results

The i-mnSod1KO mice were previously reported to show an exaggerated age-associated loss of gastrocnemius muscle mass and function (Bhaskaran et al., 2020). This was confirmed for the current cohort of old i-mnSod1KO mice where EDL force generation was reduced by ∼24% compared with age-matched WT mice (data not shown).

### Oxidation markers in nerve and muscle tissue

**Figure 1.**
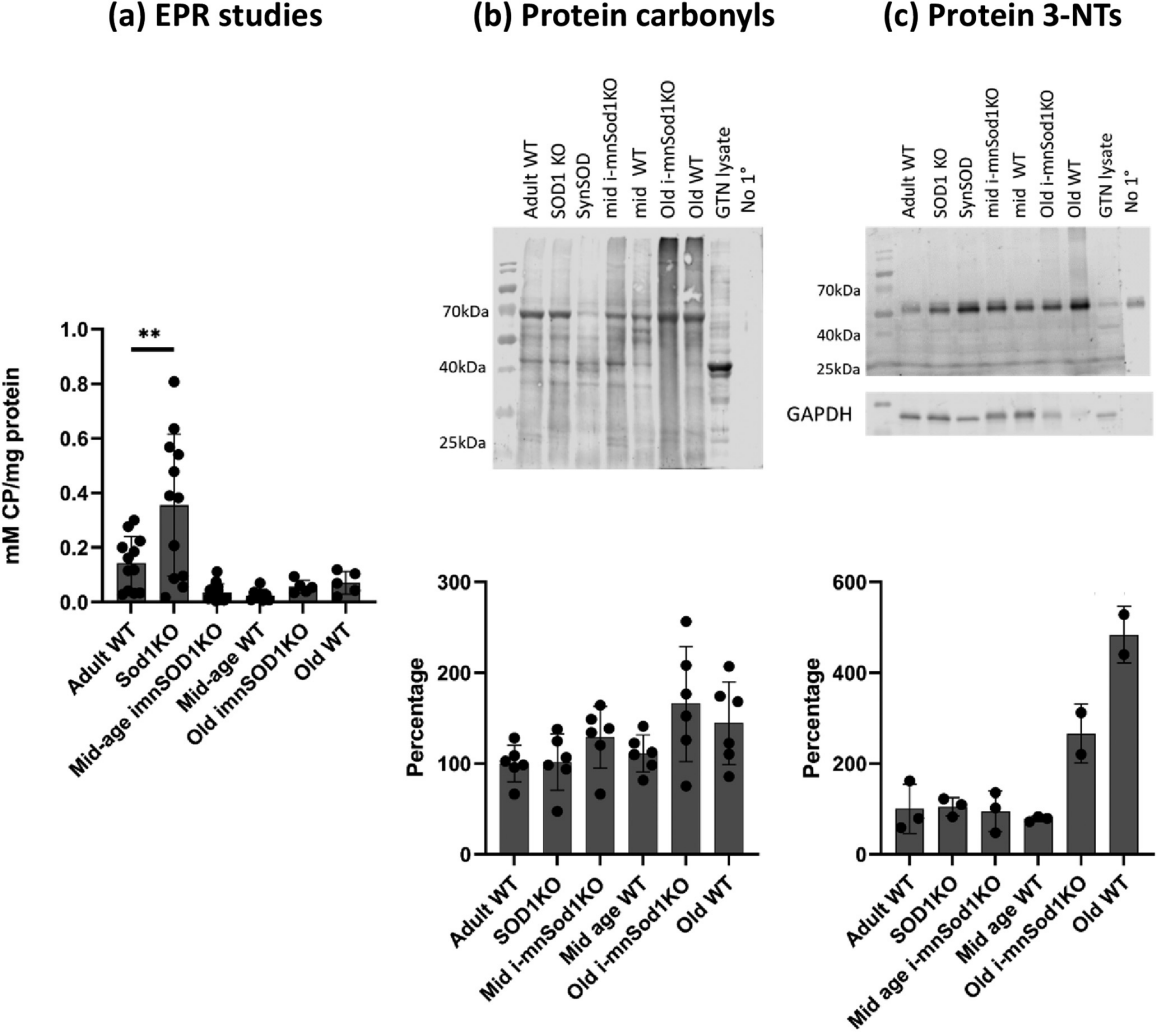
Markers of oxidation/oxidative damage in sciatic nerves of adult, mid-age and old WT, mid-age and old i-mnSod1KO and Sod1KO mice. Concentrations of CP, the EPR spin adduct in sciatic nerve (*n* = 6–14; ***a***). Representative Western blotting and quantification of protein carbonyl (***b***) and protein 3-NT (***c***) contents in sciatic nerve (*n* = 6). Densitometry of the whole lane was undertaken and data are presented as mean ± SD. Symbols represent significant differences compared with adult WT **p < 0.01, from one way ANOVA analysis with Dunnett comparison. Key: GTN-lysate = lysate of mouse gastrocnemius muscle used as a positive control sample; no 1° = lane in which no primary antibody added to determine nonspecific binding artefacts. In ***c***, only two samples were analyzed from old i-mnSod1KO and old WT mice because of lack of available sample and statistical analysis of these lanes has not been undertaken. In the original Western blottings shown nerve samples from Sod1KO mice in which Sod1 was transgenically replaced in neuronal tissue (SynSOD mice, lane 4) were also examined but these data have been reported elsewhere and are not reported here. Extended Data Figure 1-1 shows EPR analysis of the concentration of CP in skeletal muscle from the experimental groups.

The concentration of the EPR adduct, CP in sciatic nerves of adult Sod1KO mice was significantly increased compared with adult WT mice (Fig. 1*a*), but WT and i-mnSod1KO mice showed no age associated changes in the amplitude of the EPR signal. Studies of the EPR signal in the TA muscle were also undertaken and these showed an increase in the old WT mice. Muscle of Sod1KO mice also showed a significantly higher EPR signal than WT controls (Extended Data Fig. 1-1). In addition, in accordance with previously published data (McDonagh et al., 2016), the sciatic nerve was also observed to have a much higher EPR signal compared with skeletal muscle (Fig. 1*a*; Extended Data Fig. 1-1).

10.1523/ENEURO.0086-22.2023.f1-1 Extended Data Figure 1-1 EPR analysis of the concentration of CP in skeletal muscle from adult, mid-age and old WT, mid-age and old i-mnSod1KO and Sod1KO mice (*n* = 6–14). Data are presented as mean ± SD. Symbols represent significant differences (*p < 0.05, **p < 0.01) from one way ANOVA analysis with Tukey’s comparisons. Download Figure 1-1, TIF file.

Western blot analyses for protein carbonyls (Fig. 1*b*) revealed no statistically significant differences in sciatic nerves from any of the groups of KO or transgenic mice compared with WT mice either when the densitometry of the whole lane was assessed or when the four main bands were analyzed separately. Protein 3-nitrotyrosine (3-NT) levels were also examined (Fig. 1*c*). Few bands were detectable on the Western blottings of nerves for 3-NT with no evidence of any change that was specifically because of lack of Sod1 in 3NTs between the groups on raw densitometric analysis or following normalization to GAPDH content. In these latter analyses, the major band detected (∼55 kDa) was also present in the negative control and likely to be because of nonspecific binding. Thus, there was no evidence for any specific increase in oxidation in nerves of the i-mnSod1KO mice.

### Sciatic nerve morphology

**Figure 2.**
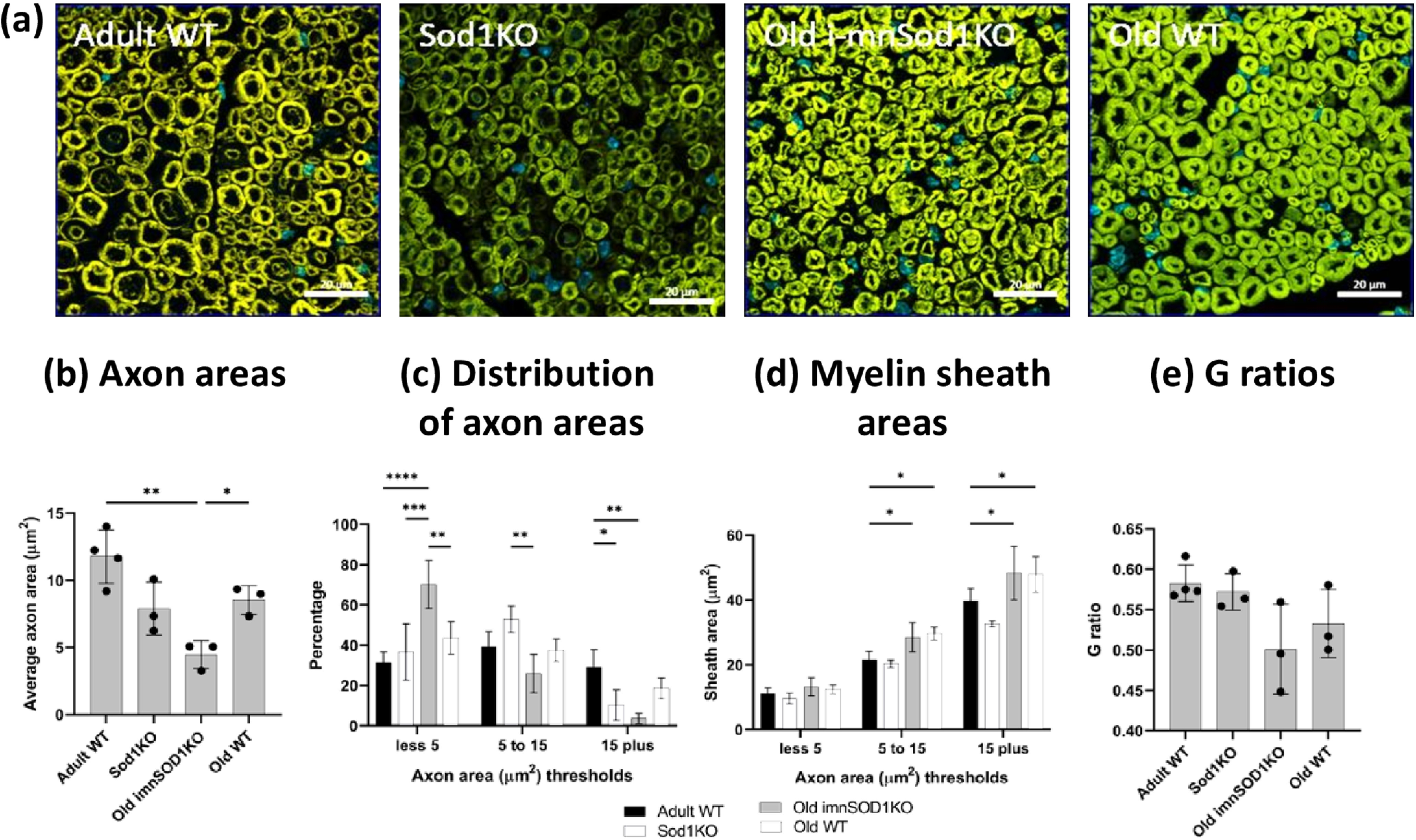
Morphology of sciatic nerves. Representative transverse cross-sections from adult and old WT mice, old i-mnSod1KO and Sod1KO mice are shown (***a***). Average axonal area (***b***), the distribution of axonal size (***c***), average myelin sheath area of axons categorized by axon size (***d***), and G-ratio (***e***) for adult, mid-age and old WT, mid-age and old i-mnSod1KO and Sod1KO mice. Scale bar = 20 μm. Data are presented as mean ± SD. Symbols represent significant differences (*p < 0.05, **p < 0.01, ***p < 0.001, ****p < 0.0001) from two-way ANOVA analysis with Tukey’s comparison (*n* = 4 nerves/group).

Representative images of transverse sections through the sciatic nerve of each experimental group are shown in Figure 2*a*. Quantitative data for individual axonal area and the area of the myelin sheath around those axons was obtained. Axonal areas are shown in Figure 2*b*, there was no significant change in axonal area in old WT compared with adult WT mice. Axons from old i-mnSod1KO were significantly reduced in area in comparison with old WT mice. The distribution in axonal areas in each group is shown in Figure 2*c* and shows that there was a significant decrease of the larger axons (>15 μm^2^) and increase in the proportion of small axons in the old i-mnSod1KO and Sod1KO mice.

The myelin areas for different sizes of axons are presented in Figure 2*d*. An increase in myelin area was seen for the larger axons in the old WT mice compared with adult mice and this was also seen in the old i-mnSod1KO mice, indicating an age-related change. The G-ratio is commonly used to assess the degree of myelination and is considered a marker of efficient signal conduction (Chomiak and Hu, 2009) and when G ratios were calculated from the above data no significant differences between groups were seen (Fig. 2*e*). In summary, these data indicate that old i-mnSod1KO mice had a decreased number of large axons and increased number of small axons compared with age-matched WT mice.

### Motor neuron quantification

**Figure 3.**
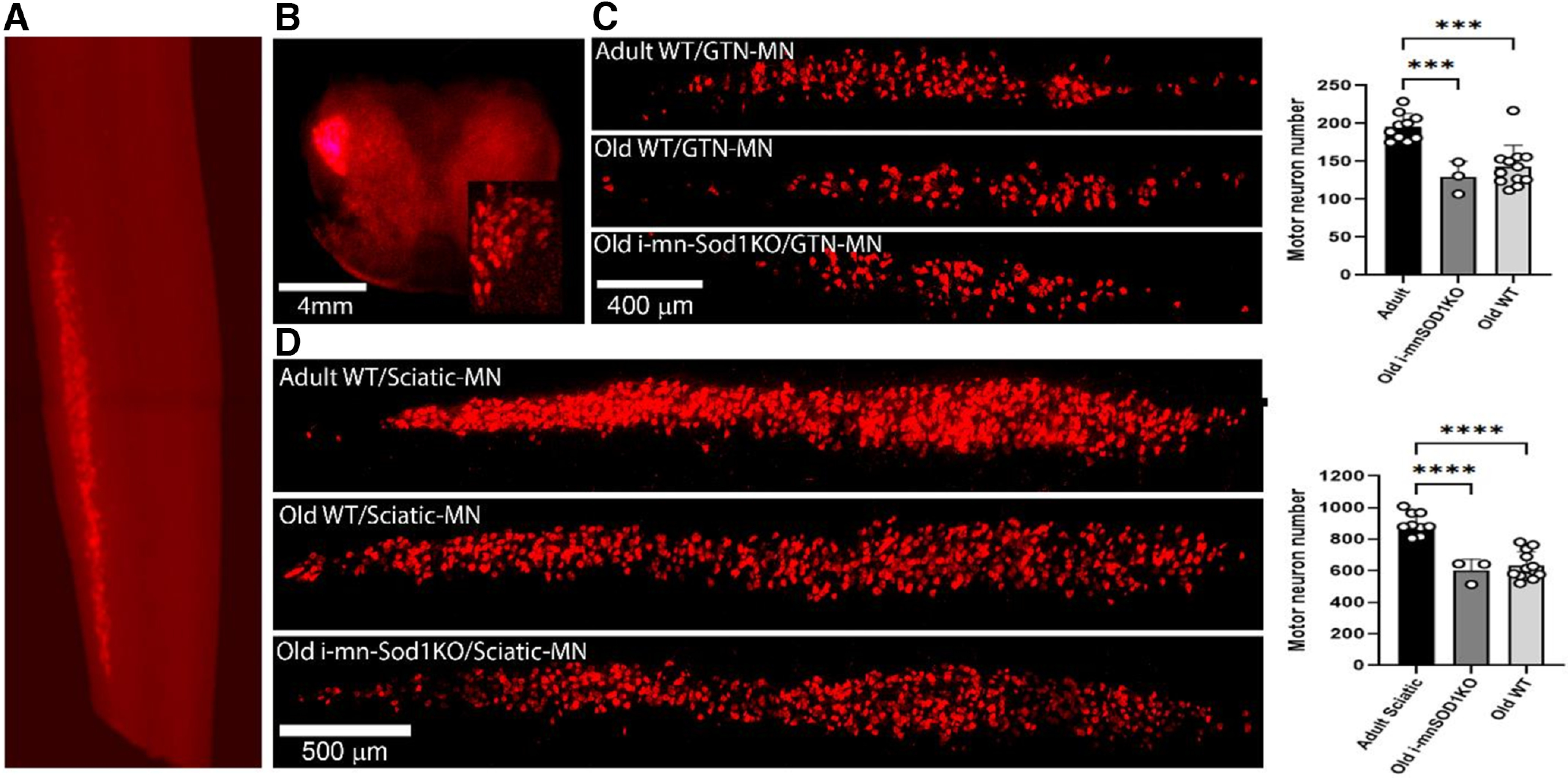
Quantification of motor neurons identified by retrograde labeling via transection of whole sciatic and the tibial nerve branches to the gastrocnemius. Representative images to show specific localization of the retrograde label to the lumbar spinal cord (***A***) and to the lateral ventral horn (***B***) after sciatic nerve transection (inset displays labeled individual motor neurons). Typical pattern of labeling of motor neurons in adult and old WT and old i-mnSOD1KO mice and quantification of numbers of labeled motor neurons following: transection of the nerve branches innervating the medial and lateral heads of the gastrocnemius (***C***), transection of the whole sciatic nerve (***D***). Data are presented as mean ± SD; ***p < 0.001 from two-way ANOVA (*n* = 3–12 animals per group).

Images illustrating the localization of the retrograde label to the lumbar spinal cord (Fig. 3*A*) and to the lateral ventral horn (Fig. 3*B*) after sciatic nerve transection demonstrate the suitability of this approach to specifically label motor neurons. We evaluated changes in motor neuron numbers specifically innervating the gastrocnemius muscle (Fig. 3*C*) and more generally for motor neurons of all the muscles associated with the sciatic nerve (Fig. 3*D*). In both situations a significant decline in the number of motor neurons was found in old WT compared with adult WT mice. The declines in axon numbers were also seen in old i-mnSod1KO mice. The percentage of motor neuron loss with age was ∼30% for both gastrocnemius specific and whole sciatic nerve evaluations, but we found no additional loss of motor neurons attributable to the lack of Sod1.

### Changes in NMJ structure

**Figure 4.**
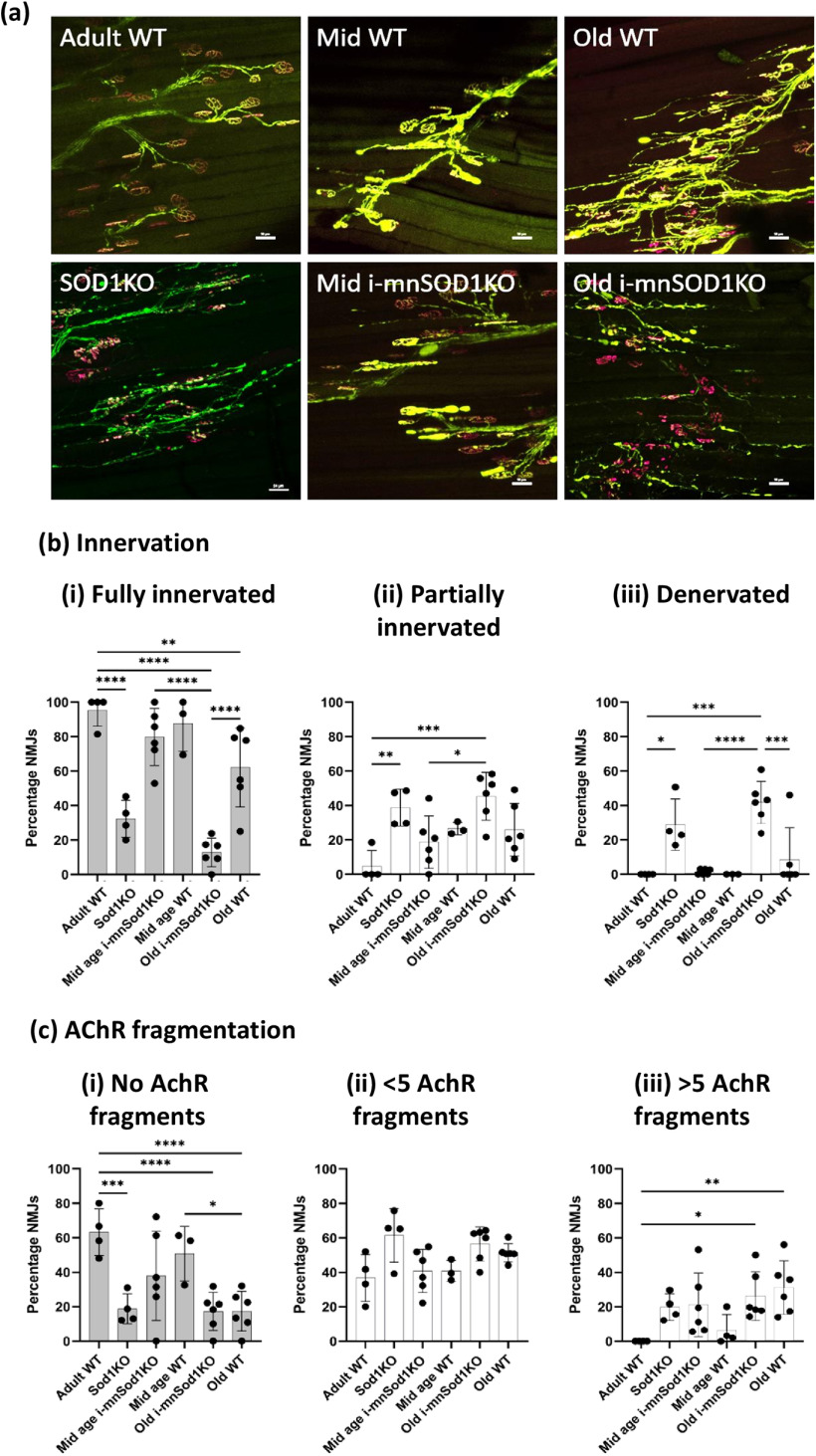
Morphologic assessment of NMJ innervation and fragmentation. Representative images of NMJ and axonal structure seen following bungarotoxin staining (magenta) and Thy1-YFP/CFP (green) visualization in whole EDL muscles from adult, mid-age and old WT, mid-age and old i-mnSod1KO and Sod1KO-Thy1-CFP mice; scale bar = 50 μm (***a***). Extent of innervation assessed by percentage overlap of presynaptic nerve terminal and AchR categorized as fully innervated (i), partially innervated (ii) or denervated (iii; ***b***). Average number of AchR fragments reported as none (fully intact; i), <5 (ii), >5 fragments (iii; ***c***). Data are presented as mean ± SD. Symbols represent significant difference between groups (*p < 0.05, **p < 0.01, ***p < 0.001, ****p < 0.0001) from two-way ANOVA with Tukey’s multiple comparison (*n* = 4–6 muscles per group).

Images of NMJs from EDL muscles were captured via confocal microscopy to allow detailed examination of their structure. Figure 4*a* provides representative examples from z-stacked images of NMJ from adult, mid-age and old WT mice, Sod1KO mice and mid-age and old i-mnSod1KO mice. In these images the AchR clusters were stained with bungarotoxin and the nerve visualized via CFP or YFP expression. Axonal organization is clear and defined in adult WT but is highly disordered in both old WT and old i-mnSod1KO mice.

The average area of the muscle fiber occupied by individual NMJs showed no significant alteration either with advancing age or between any of the knock-out mouse models (data not shown). Overlap of presynaptic and postsynaptic regions was quantified and is shown in Figure 4*b*, i–iii. Fully innervated NMJ had complete overlap, partially innervated NMJ meant that some of the AchRs were visible without YFP overlay and in fully denervated only AchRs were visible. The values are expressed as a percentage of the total number of NMJs counted per muscle. NMJs in muscle from adult WT mice were effectively fully innervated. NMJs from Sod1KO mice were found to be variable with roughly equivalent proportion innervated, partially innervated and denervated. At mid-age the WT and i-mnSod1KO mice had very similar profiles and showed some partially or fully denervated NMJs, but the changes were not significantly different to WT adult mice. There was a significant loss of fully innervated NMJs in both old WT (*p* = 0.0071) and old i-mnSod1KO (*p* < 0.0001), but this loss was significantly greater in the old i-mnSod1 mice with <15% of NMJ remaining fully innervated [Fig. 4*b*(i)].

Fragmentation of the NMJ endplates was quantified [Fig. 4*c*(i–iii)] by counting the number of AchR clusters that were clearly separate and these were categorized as previously (Staunton et al., 2019). An intact endplate was recorded where no fragments were seen, this accounted for almost 70% of NMJs in adult WT mice [Fig. 4*c*(i)]. The mid-aged WT and mid-aged i-mnSod1KO mice were not significantly different to each other or to adult WT mice with respect to the number of intact endplates. There was a significant decline in intact endplates in both old WT and old i-mnSod1KO mice compared with adult WT (*p* < 0.0001) and their own mid-age groups. This decline in intact endplates in both of the old groups was associated with a significant increase in the highly fragmented (more than five fragments) endplates observed in both groups [Fig. 4*c*(iii)]. There were no significant differences between the age-matched groups in any of the categories indicating there was no specific effect on AchR fragmentation that could be attributed to the lack of Sod1 rather than the age of the mice.

**Figure 5.**
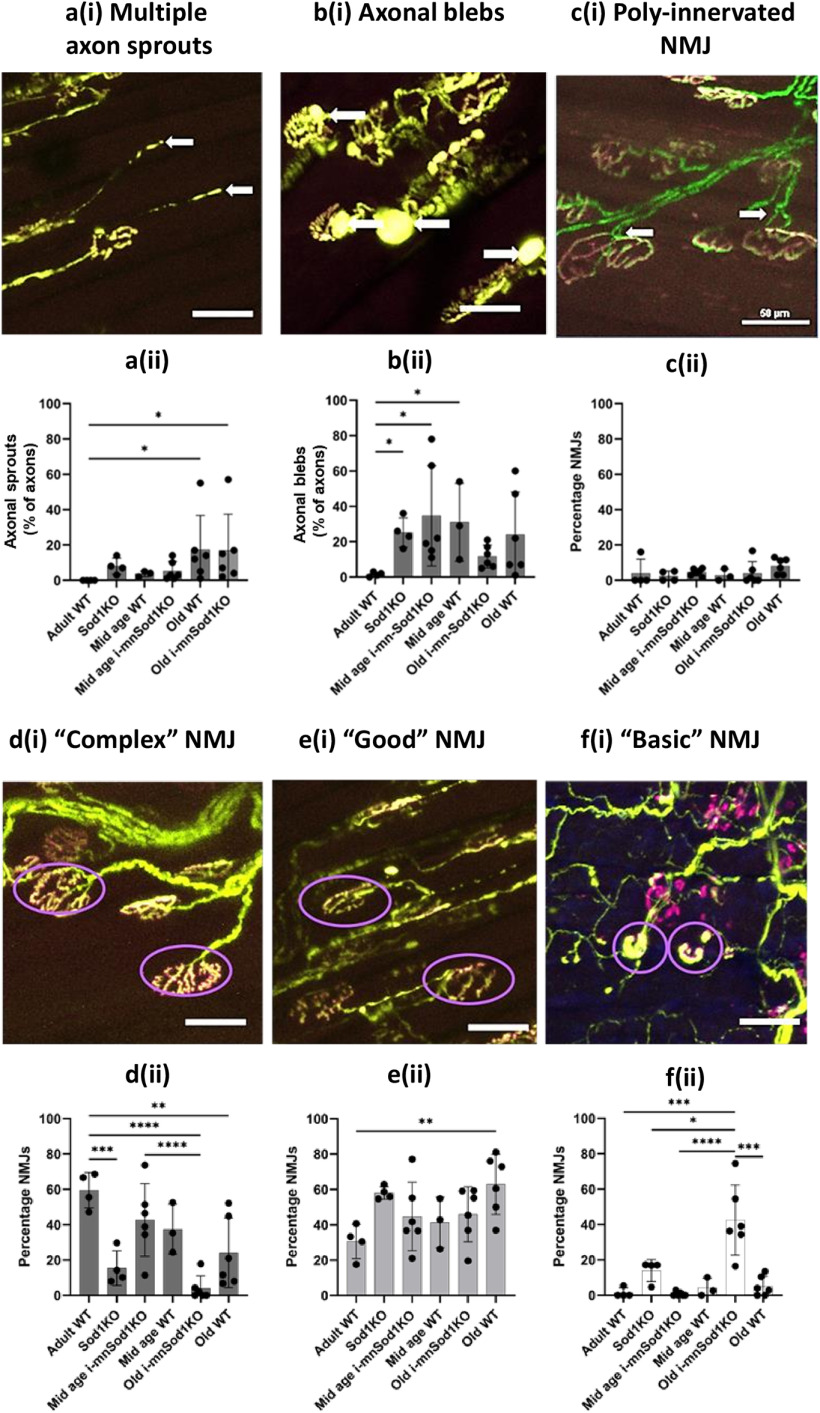
Quantification and representative images of specific NMJ structural alterations in EDL muscle from adult, mid-age and old WT, mid-age and old i-mnSod1KO and Sod1KO-Thy1-CFP mice. As in Figure 4, axons are shown in green and NMJ in magenta. An example of axons with multiple sprouts at ***a***(i) with percentage of axons having multiple sprouts, ***a***(ii). Examples of axons with multiple blebs [***b***(i)] with the percentage of axons having blebs [***b***(ii)] and a representative image of poly innervation of NMJ at ***c***(i) with the percentage of NMJs which show evidence of multiple innervations at ***c***(ii). Complexity of the NMJ was categorized as complex [***d***(i) and ***d***(ii)], good [***e***(i) and ***e***(ii)], or basic [***f***(i) and ***f***(ii)]. Representative images of complex [***d***(i)], good [***e***(i)], and basic [***f***(i)] NMJ are shown with the specific NMJ displaying the characteristics circled in purple. Data are presented as mean ± SD. Symbols represent significant differences between groups based on two-way ANOVA with Tukey’s comparison (*n* = 4–6 muscles per group); *p < 0.05, **p < 0.01, ***p < 0.001, ****p < 0.0001.

Sprouting of axons and the presence of blebs on the axons were assessed. Figure 5*a*(i) shows an example image illustrating multiple sprouting axons and Figure 5*a*(ii) shows the percentage of axons in which sprouting was evident. There was no evidence of sprouting in the adult WT groups but the percentage of sprouts increased significantly in the old WT and i-mnSod1KO groups of mice. An example image illustrating multiple axonal blebs is shown in Figure 5*b*(i) and the percentage of axons having blebs in each group is shown in Figure 5*b*(ii). A significant increase in the percentage of axons showing blebs was seen in the Sod1KO mice and in both mid-aged WT and i-mnSod1KO mice with no significant increase in the old groups.

Multiple innervation of single NMJs is also occasionally seen in samples from older mice and this was examined in the groups. Figure 5*c*(i) shows an example image illustrating multiple axonal innervation of a NMJ and Figure 5*c*(ii) shows the percentage of NMJ with multiple axonal innervation. No differences were seen between the groups. Thus, there were no specific changes in axonal sprouting, axonal blebs or poly-innervation of NMJ in the i-mnSod1KO group which could not be attributed to the age of the mice.

Images of NMJ show different levels of complexity with significant variability from the “pretzel shape” classically reported. A scoring system was developed to attempt to quantify and understand whether Sod1KO affected the complexity of the NMJ structure. These ranged from “complex” NMJ with numerous convolutions compared with those which were simply disks with little evidence of internal structure (“basic”). An intermediary level of complexity (“good”) was also used. Examples of “complex,” “good,” and “basic” NMJ are shown in Figure 5*d*(i),*e*(i),*f*(i), and the values obtained from each group are shown in Figure 5*d*(ii)–*f*(ii). Approximately 60% of NMJs in adult WT mice had complex junctions with <2% categorized as “basic.” Old i-mnSod1KO mice had the most striking changes with ∼40% of NMJs considered to be “basic,” this was a significant increase compared with adult WT (*p* < 0.0001), old WT (*p* = 0.0003), and mid-age i-mnSod1KO (*p* < 0.001). The old i-mnSod1KO (*p* < 0.0001) and Sod1KO (*p* = 0.0051) mice showed a significant decrease in the percentage of “complex” NMJs compared with adult WT. i-mnSod1KO mice therefore showed a significant switch to NMJ having a simple disk structure (i.e., “basic” structure) compared with age-matched WT mice indicating a specific effect of lack of Sod1.

## Discussion

Previous studies examining the i-mnSod1KO mouse demonstrated a significant reduction of Sod1 expression in brain, spinal cord and sciatic nerve and concluded that neuron-specific deletion of CuZnSOD is sufficient to cause motor neuron loss in young mice, but loss of innervation may not be sufficient to induce muscle fiber loss until the muscle reaches a threshold beyond which it cannot compensate for neuronal loss (Bhaskaran et al., 2020). Our aim here was to define the way in which a lack of *S*od1 affects neuronal tissue to precipitate the premature age-related loss of muscle mass. Data obtained indicate that by old age, i-mnSod1KO mice showed increased numbers of fully denervated NMJ, a reduced number of large axons and increased number of small axons compared with age-matched WT mice. This was associated with changes in the morphology of the remaining innervated NMJ, which predominantly displayed a less complex structure. In contrast multiple other changes in the nerves and NMJ of old i-mnSod1KO mice (increased protein 3NT content, increased myelin sheath areas, reduced axonal number and fragmentation of NMJs) were also observed in the nerves of old WT mice and appear to be related to aging.

A number of measures of oxidation were examined to determine whether the lack of Sod1 in nerve led to increased oxidation. We previously reported (Sakellariou et al., 2014; McDonagh et al., 2016) that 3-NT and protein carbonyls in nerves did not show a significant increase in old WT mice, Sod1KO mice, nSod1KO or mSod1KO mice, although elevated protein carbonyls in the sciatic nerve of Sod1KO mice has been reported by others (Hamilton et al., 2013). No effect on nerve protein carbonyl contents was seen here, although there was a tendency for nerve protein 3-NT content to increase in both old i-mnSod1KO and old WT mice compared with younger groups. Increased protein 3-NT residues are a mark of oxidation by peroxynitrite which was previously proposed as a mechanism for tissue oxidation in Sod1KO mice (Sakellariou et al., 2011). Tissues were also examined using an EPR technique which previously indicated increased oxidation of the CPH probe in tissues from old compared with adult WT mice (McDonagh et al., 2016). The probe reacts preferentially with superoxide and peroxynitrite and an age-related increase in muscle from WT mice was seen (Extended Data Fig. 1-1). An increase was also seen in the sciatic nerve of Sod1KO mice, but no changes in the nerves of old WT or old i-mnSod1KO mice were detected. Thus, we find no evidence that nerves of i-mnSod1KO mice show an increase in oxidation above that seen in age-matched mice and hence that the premature changes in nerve and NMJ structure and muscle loss are unrelated to increased nerve oxidation. This lack of evidence for major changes in markers of oxidative damage in nerves of i-mnSod1KO mice is compatible with our previous conclusion that the effect of lack of Sod1 in nerves results in impaired redox signaling, rather than oxidative damage, which plays a key role in muscle loss in Sod1KO mice (Sakellariou et al., 2018). Bhaskaran et al. (2020) reported an increased marker of oxidation (F2-isoprostanes) in muscle of old i-mnSod1KO mice compared with old WT mice, but no studies of nerve oxidation were reported.

The previous study of i-mnSod1KO mice (Bhaskaran et al., 2020) examined motor neuron numbers in the ventral spinal cord of mice and reported an estimated early loss of motor neurons in i-mnSod1KO mice which also occurred with age in WT mice. We have used a retrograde labeling approach which is specific to individual nerves to examine motor neuron numbers. The total number of retrograde labeled sciatic nerve-associated motor neurons in adult mice is remarkably similar to that reported previously (Žygelytė et al., 2016), suggesting that it is a robust method for determining changes in motor neuron numbers. The data showed a substantial (∼30%) decline in motor neurons with age for both retrograde gastrocnemius/soleus specific and whole sciatic nerve evaluations which was not exacerbated in the i-mnSod1KO mice (Fig. 3). While this technique does not differentiate between motor neuron subpopulations, α motor neurons make up the largest percentage of efferent neurons innervating a lower limb muscle (Burke et al., 1977; Mierzejewska-Krzyżowska et al., 2019).

Evidence that the lack of Sod1 affected axonal integrity was seen in a loss of axonal area in individual axons of the sciatic nerve of old i-mnSod1KO mice compared with old WT mice. These changes appeared because of a reduction in the proportion of larger axons and a greater proportion of small axons in i-mnSod1KO mice in comparison with adult and old WT (Fig. 2). Together with the lack of decline in total axonal number reported above (Fig. 3), these data appear to indicate an effect of Sod1 in reducing the area of larger axons (and hence increasing the number of smaller axons).

The sciatic nerve also showed an increase in myelin thickness with advanced age as previously reported in other nerves (Peters, 2002). Both old i-mnSod1KO and age matched WT showed increased sheath thickness compared with adult mice. The G-ratio (ratio of the inner axonal diameter to the total outer diameter) is a functional and structural index of optimal axonal myelination (Rushton, 1951; Chomiak and Hu, 2009), but no significant changes were seen between the groups studied. A decreased G-ratio with advancing age has been described in rats (Azcoitia et al., 2003; Amer et al., 2014).

We evaluated in detail the innervation of NMJ, fragmentation of AchRs, numbers of axonal sprouts and blebs, poly-innervation of NMJ, and complexity of the NMJ structure (Figs. 4 and 5). Aging caused loss of innervation, increased fragmentation, increased axonal sprouting and led to a reduced number of complex NMJs (which showed a much simpler structure). A lack of neuronal Sod1 greatly exacerbated the loss of NMJ innervation in older mice and caused an increased proportion of the NMJ to show a simpler structure, but NMJ fragmentation and axonal sprouting were unaffected by lack of Sod1. Both old WT and old i-mnSod1KO mice showed comparable changes in NMJ fragmentation, but comparison with muscle force production indicates that fragmentation may have a limited influence on signaling for force production. Furthermore, it also suggests that a lack of Sod1 in motor neurons has little influence on the postsynaptic complexes that help to maintain NMJ structure.

The lack of neuronal Sod1 in old i-mnSod1KO mice led to accelerated changes in the complexity (pretzel shape) of NMJs with age. Thus, a significant percentage of NMJs in old i-mnSod1KO mice showed similar structure to embryonic (simple fragments) rather than mature pretzel shaped NMJs. It has been shown (Kang and Lichtman, 2013) that AchR clusters are lost if not re-innervated and the increased presence of NMJs with a pseudo-embryonic structure may reflect a lack of re-innervation in i-mnSod1KO mice which also showed a significant decline in NMJ innervation, in comparison with adult and old WT mice.

Thus, our data indicate that a lack of Sod1 in neuronal tissue leads to an accelerated age-related loss of skeletal muscle mass and function by accelerating or exacerbating a number of the many changes that occur in motor neurons and NMJ with aging. Although previous studies indicated that i-mnSod1KO mice showed loss of motor neurons in the ventral spinal cord compared with that of WT mice (Bhaskaran et al., 2020), we found no additional loss of motor neurons in excess of that which occurs in old WT mice. The assessments of motor neuron numbers in this study and that of Bhaskaran et al. (2020) used different approaches, both of which are likely to have limitations that may have contributed to this divergence. For example, direct counting of motor neurons in sections of the ventral spinal cord may be limited in the ability to detect small motor neurons, while the retrograde labeling approach requires all axonal termini to be accessible to the label and the axons capable of retrograde transport. Lack of neuronal Sod1 led to a greater number of small axons and a reduced number of large axons in the sciatic nerve of old i-mnSod1KO mice in comparison with old WT. Such changes were associated with a substantial reduction in the number of fully innervated NMJ and an increase in the number of denervated NMJ in the i-mnSod1KO mice, while remaining AchR also showed a reversion to an embryonic-type structure. Thus, the data obtained indicate that lack of Sod1 specifically in motor nerves may cause premature muscle atrophy through inducing changes in axonal area, reduction in the relative size of axons, greater overall denervation and modification of NMJ to a less complex structure. It is interesting to speculate why deletion of Sod1 in motor nerves has a greater effect on synaptic structure and integrity in comparison with the effects on the motor neuron cell body. Sod1 plays a well-established role in control of redox signaling and prevention of oxidative damage. The predominance of effects of the deletion in the motor neuron periphery suggests that components of the motor neuron periphery have a greater requirement for controlled redox signaling, or that local prevention of oxidative damage is particularly critical. Whether this relates to differences in the relative dependence on mitochondrial respiration or the overall capacity to prevent oxidative damage is currently unknown.

The question remains as to why the changes in nerve structure and function induced by lack of Sod1 only present as a sarcopenic phenotype in older mice, although the inducible loss of Sod1 in neuronal tissue was reported from 10 months of age. The likely explanation is that appearance of any muscle phenotype is delayed because of expansion of the size of motor units with collateral re-innervation of denervated NMJ. The time delay therefore reflects the period until the ability to expand motor unit size is exceeded. While motor units have a capacity for expansion to compensate for motor neuron loss, this process may modify and compromise aspects of synaptic transmission (Mantilla and Sieck, 2009; Thomas et al., 2014) and there appears to be a maximum ability to increase the size of an individual motor unit (Thompson and Jansen, 1977). A 25–50% reduction in the number of motor neurons is reported to occur in both man and rodents with aging (Tomlinson and Irving, 1977; Rowan et al., 2012). In humans, Piasecki and colleagues showed that motor unit loss occurred early in the aging process and that failure to expand was characteristic of sarcopenia (Piasecki et al., 2018). Our results are consistent with motor unit loss contributing to decreased force generation with age that is exacerbated by lack of neuronal Sod1 although in both situations the initial loss of neuromuscular integrity is likely to have been mitigated by an ability to expand the size of motor units.

## Synthesis

Reviewing Editor: Eran Perlson, Tel Aviv University Decisions are customarily a result of the Reviewing Editor and the peer reviewers coming together and discussing their recommendations until a consensus is reached. When revisions are invited, a fact-based synthesis statement explaining their decision and outlining what is needed to prepare a revision will be listed below. The following reviewer(s) agreed to reveal their identity: NONE. Note: If this manuscript was transferred from JNeurosci and a decision was made to accept the manuscript without peer review, a brief statement to this effect will instead be what is listed below.

he manuscript “Deletion of Sod1 in motor neurons exacerbates age-related changes in axons and NMJs associated with premature muscle atrophy in aging mice” is an interesting article whose main aim was to “examine the effects of age and lack of Sod1 specifically in neurons on motor neuron number and explore the changes in nerve oxidation status as well as structural changes in axons and NMJs”. The authors describe the effect of SOD1 deletion in the motor neuron of aged mice at the cell body, axon, and NMJs. They also use biochemical assays to assess the effects of SOD1 deletion on oxidative damage in the motor neuron periphery.

Although the work contains interesting data and a better understanding of SOD1 effects on age and oxidative challenge on motor neuron different compartments is timely, the provided data is very similar to previous publications by the same authors and is purely descriptive without the addition of a new hypothesis or testing of novel mechanisms. Furthermore, data presentation is inadequate and necessitates further work making it premature for publication at this stage.

Specific points are listed below:

1. The authors should be very clear on what are the exact differences between their results here with those already published (particularly Bhaskaran et al., Aging Cell 2020).

In Bhaskaran et al. (Aging Cell 2020), most differences were detected by comparing age-matched controls, as expected. However, the current manuscript highlights differences between the “Adult WT” group and the “Old imnSOD1KO” which are not statistically evident in the “Old WT” group. What is the biological relevance of such a comparison? The authors should separate the description of the data when comparing Adult v/s Old animals (indeed, some results are inconsistent with previous findings; see below), which is different than when comparing WT v/s animals with altered expression of SOD1. In other words, the missing statistical comparison between “Old imnSOD1KO” and “Old WT” is the most relevant for this work.

Why old animals do not exhibit oxidative stress, as expected, and as already published (Bhaskaran et al., Aging Cell 2020)? Please discuss.

2. The way data are presented is confusing, mainly because some differences between experimental groups are due to age (most of these differences have been already published) but not to the absence of SOD1 in neurons.

Figure 1 - The hypothesis described by the authors is that lack of neuronal SOD1 is sufficient to induce age-related muscle atrophy and NMJ disruption that does not occur by reduced oxidation. However, the data presented here do not provide strong evidence to support it. The authors claim that the lack of SOD1 does not cause reduced oxidation based on several biochemical analyses that are showing confusing evidence. In figure 1b, there is a lack of a loading control for protein carbonyl, and there are many unspecific bands which makes it difficult to determine the reliability of this result. In figure 1c, please indicate the band whose intensity was measured and plotted. The intensity of the loading control is very uneven between lanes. This lack of uniformity could explain some of the quantifications. The last lane (Nº 1) displays an abrupt decrease in the background. If this is not a single membrane I would recommend displaying the last lane separately. Thus, it is hard to draw conclusions in regard to the mechanism by which SOD1 is influencing motor neuron oxidative stress based only on this evidence. I would suggest to try and use essays to analyze mitochondrial structure or activity to further strengthen their claims in addition to adding more representative blots.

3. Figure 2 - It is hard to draw conclusions based on the presented images. There is no scale bar, no markings to show what the florescent colors stand for, no high magnification images of single axons. There is a need also to show the image of myelin together with axonal marker. The graphs need to be separated by age groups. It would be better to present axon diameters and sheath area in a histogram of frequencies rather than the current shape.

. In Fig. 2a:

- replace pictures where DAPI staining is not evident (e.g. Adult WT).

- order pictures in the same order as bars of the plots.

- double-check that pictures and bars within the plots are correct. For instance, Old imnSOD1KO axons look thicker than those of old WT mice, but the plot shows the opposite. Also, SynSOD axons look similar (or even thicker) than Adul WT ones, opposite to the plot.

Figure 3 - The images are oversaturated, making it is very hard to see single motor neurons. What are the images in 3c-3d? (include a representative panel of the “Old imnSod1KO” condition in panels 3c and 3d.); are they part of a spinal cord section or a piece of peripheral nerve? It is unclear why the authors chose to do two types of axon resection rather than to count the total number of motor neurons in the spinal cord sections as was done in Bhaskaran et al (2020). Also, the result in this figure is inconsistent with Bhaskaran et al. Data in Bhaskaran et al. (Aging Cell 2020) showed less MN somas in the “Old imnSod1KO” group than in the “Old WT group”. The explanation discussed in the current manuscript is technical i.e. that small motoneuron somas may not have been manually counted. This explanation is hard to conceal as even small somas (how small they could be?) should be stained and hence quantified.

The authors must address this experimentally, best by performing a similar analysis to Bhaskaran et al or to provide further explanation for this discrepancy. If there is no significant difference in cell numbers it will be interesting to make sections of the sciatic nerve in several positions (distal and proximal) to assess if the denervation process stops at the level of the NMJ or axonal numbers are decreased as well, showing a dying back phenomenon.

4. Figure 4 - Please describe the exact scale bar in the figure legend, the text in the image is too small. The visualization of the graphs makes it difficult to discriminate between age-related changes and SOD1 KO effect. Can the authors provide further explanation for the difference between 3b and 3c in regard to two old mice groups?

. In Fig. 4a:

- please improve the quality of the pictures. at least the ones in the manuscript I got seem of very low quality and with very uneven intensities.

- enlarge at least one size bar and its value to make it clearer.

- please consider replacing green/red combinations for the color-blind.

- add insets with higher magnified pictures to better see what is described.

. In Fig. 4b:

- why only the fully innervated group was compared? evaluating potential differences between age-matched partially innervated and denervated groups could be more biologically relevant.

- the text does not mention/discuss the differences between the “Old i-mnSod1KO” and “Old WT” which are observed in the graph.

- please double-check the statistical differences regarding the comparison of old with adult mice (line 271).

5. In Fig. 5:

- please define “sprouting” and add arrows/arrowheads in panel 5a(ii) to indicate them.

- based on panel 5c(ii), it is possible that NMJs counted as having multiple innervations may in fact be singly innervated by an axon that branched near the postsynaptic region. how far each axon was traced from each innervating NMJ?

- regarding data from panels of Fig. 5d, standard classifications of postsynaptic morphology in different conditions have been published in several articles. In the absence of a clear definition of what is called “good” or “fair” NMJs, it will be worth using already published categories. For instance, “complex” and “basic” have been described previously in many works as “pretzel” and “plaques”. In this regard, why the “fragmented” morphology (an expected shape for old NMJs) was not considered/quantified?

6. General comments -

a. Please replace bar graphs with scatter plots (with or without bars) as they allow a better assessment of data dispersion. Please add clear-size bars to all panels.

b. please provide visual separation of the groups by age in all graphs. Age matching is critical in this work as its aim is to point to the age-related differences due to SOD1 deficiency.

c. The use of mice models of different ages and genotypes is confusing. For example, there is use of SynSod model only in figures 1 and 2, in figure 2 there are no SOD1KO samples, mid-age mice are only used in figures 4-5. Please provide an explanation for this discrepancy or add the missing models to provide consistency.

d. Graphs should show data distribution, preferably using individual values if n<10.

e. The authors need to state the exact n and what it stands for each graph.

f. The writing of the manuscript needs to be improved. It is hard to understand the conclusions the authors drew from their results as they are written in a technical and descriptive manner, without any connection between them and the main hypothesis. I would suggest including an additional sentence in each paragraph that will explain in their words what insight we gain from each dataset.

In supplementary Fig. 2:

please discuss how the deletion of SOD1 in neurons could result in augmented oxidative stress in old muscles? please also discuss why this does not happen in the imnSOD1KO mice.

- when discussing motor units and their limitations for synaptic transmission, please include more references, as this very relevant issue has been extensively discussed.
