# Supplementary figures and images for: Deletion of Sod1 in Motor Neurons Exacerbates Age-Related Changes in Axons and Neuromuscular Junctions in Mice
Source: eNeuro. 2023 Mar 10;10(3):ENEURO.0086-22.2023. doi: 10.1523/ENEURO.0086-22.2023 (PMC10026931; doi:10.1523/ENEURO.0086-22.2023)

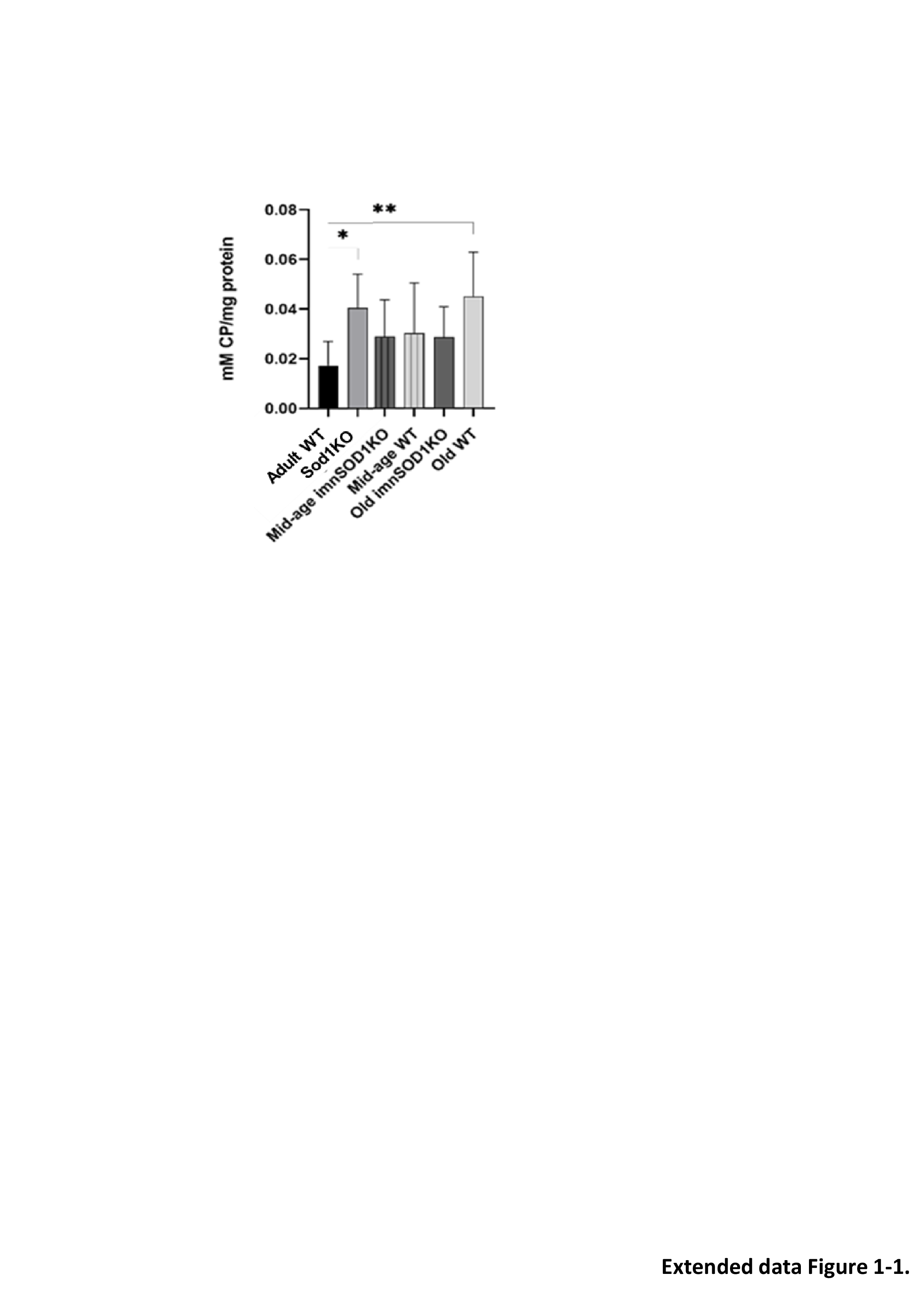

Supplement: Extended Data Figure 1-1 — EPR analysis of the concentration of CP in skeletal muscle from adult, mid-age and old WT, mid-age and old i-mnSod1KO and Sod1KO mice (n = 6–14). Data are presented as mean ± SD. Symbols represent significant differences (*p < 0.05, **p < 0.01) from one way ANOVA analysis with Tukey’s comparisons. Download Figure 1-1, TIF file. [file enu-eN-NWR-0086-22-s01.tif]
